# Evaluation of the Maternal Death Surveillance and response system in Hwange District, Zimbabwe, 2017

**DOI:** 10.1186/s12884-019-2255-1

**Published:** 2019-03-29

**Authors:** Mpumelelo Maphosa, Tsitsi P. Juru, Nyasha Masuka, More Mungati, Notion Gombe, Peter Nsubuga, Mufuta Tshimanga

**Affiliations:** 1 Department of Community Medicine, University of Zimbabwe, Health Studies Office, P. O Box CY 1122 Causeway, Harare, Zimbabwe; 2 Ministry of Health and Child Care, Matabeleland North Province Harare, Zimbabwe; 3 Elizabeth Glazer Paediatric AIDS Foundation, Mbabane, Swaziland; 4 Global Public Health Solutions, Atlanta, USA

**Keywords:** Evaluation, Maternal Death Surveillance and response, Hwange, Zimbabwe

## Abstract

**Background:**

Maternal Death Surveillance and Response (MDSR) system was established to provide information that effectively guides actions to eliminate preventable maternal mortality. In 2016, Hwange district sent six maternal death notification forms (MDNF) to the province without maternal death audit reports. Timeliness of MDNF reaching the province is a challenge. Two MDNF for deaths that occurred in February and May 2016 only reached the provincial office in September 2016 meaning the MDNF were seven and four months late respectively. We evaluated the MDSR system in Hwange district.

**Methods:**

A descriptive cross-sectional study was conducted. Health workers in the sampled facilities were interviewed using questionnaires. Resource availability was assessed through checklists. Epi Info 7 was used to calculate frequencies, means and proportions.

**Results:**

We recruited 36 respondents from 11 facilities, 72.2% were females. Inadequate health worker knowledge, lack of induction on MDSR, unavailability of guidelines and notification forms and lack of knowledge on the flow of information in the system were reasons for late notification of maternal deaths. Workers trained in MDSR were 83.8%. Only 36.1% of respondents had completed an MDNF before. Respondents who used MDSR data at their level were 91.7%, and they reported that MDSR system was useful. Responsibility to complete the MDNF was placed on health workers. Maternal death case definitions were available in 2/11 facilities, 4/11 facilities had guidelines for maternal death audits. It costs $60.78 to notify a maternal death.

**Conclusion:**

Reasons for late notification of maternal deaths were inadequate knowledge, lack of induction, unavailability of guidelines and notification forms at facilities. The MDSR system is useful, acceptable, flexible, unstable, reliable but not simple. Maternal case definitions and maternal death audit guidelines should be distributed to all facilities. Training of all health workers involved in MDSR is recommended.

## Background

Maternal Death Surveillance and Response (MDSR) refers to continuous, systematic collection, analysis, interpretation and dissemination of data regarding maternal deaths. It links the health information system and quality improvement processes from local to national levels [1]. The World Health Organization (WHO) defines a maternal death, as the death of a woman while pregnant or within 42 days of termination of pregnancy. This is irrespective of the duration and the site of the pregnancy, from any cause related to or aggravated by the pregnancy or its management but not from accidental or incidental causes [2–4].

In 2015, the maternal mortality ratio was estimated at 216/100,000 live births globally [5]. This maternal mortality ratio translates to approximately 830 women dying every single day due to the complications of pregnancy and childbirth. Almost all these deaths occurred in low resource settings, and most could have been prevented [6, 7]. The WHO African Region bore the highest burden with almost two-thirds of global maternal deaths occurring in the region [8]. The burden is more pronounced in poor, rural areas where young adolescents face a higher risk of complications and death as a result of pregnancy [9]. In Zimbabwe, according to WHO the maternal mortality ratio was 614/100000 live births in 2014 [10]. The Millennium Development Goal (MDG) 5 of reducing the maternal mortality ratio to 71/100000 was far from being achieved [11]. Like many other developing nations, Zimbabwe failed to achieve the target for MDG 5 by 2015.

**Fig. 1:**
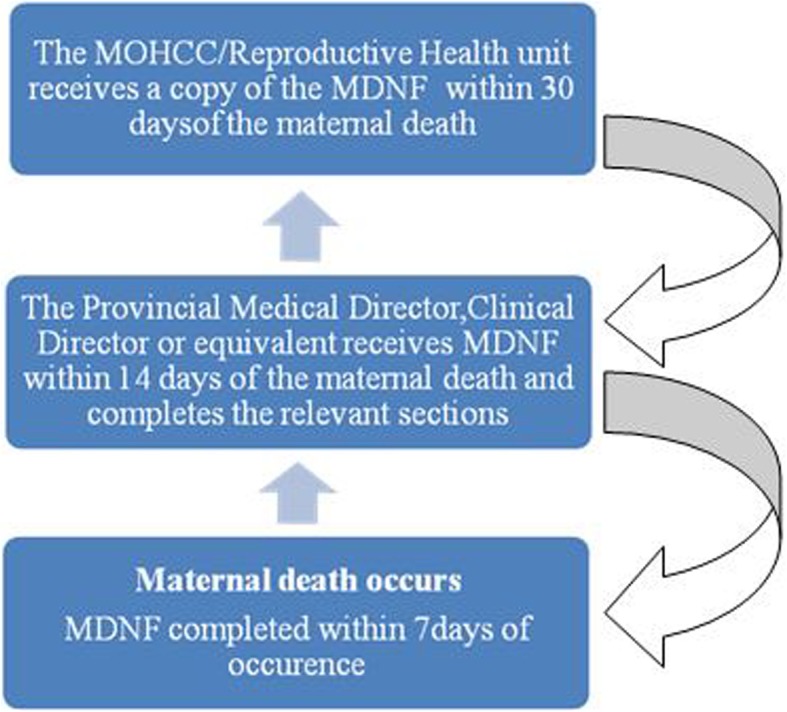
Current flow of the Maternal Death Notification Form in Zimbabwe

In the early 1990s, Zimbabwe established a Maternal Mortality Surveillance and Response (MMSR) with the aim of providing information that effectively guides actions to eliminate preventable maternal mortality. In 2013 the name of the surveillance system was changed to MDSR in line with the WHO guide of ending preventable maternal mortality [12]. The Sustainable Development Goals proposed that progress toward ending preventable maternal deaths should continue to be measured by monitoring the Maternal Mortality Ratio [13]. When a maternal death occurs, three copies of the maternal death notification form are completed, and one form is retained at the facility where the death occurred. Two forms are then transmitted to the district for capturing into the District Health Information System (DHIS 2) then the two forms are sent to the province within 14 days of the death. At the province, the Provincial Maternal and Child Health Officer completes the relevant sections and retains one copy. The last copy of the form is then submitted to the Reproductive Health unit at the head office within 30 days of the occurrence of a maternal death. Feedback is given at each level of the health care system [Fig. 1].

In 2016, Matabeleland North province recorded 24 maternal deaths compared to 17 deaths in 2015. Of the 24 deaths recorded, Hwange district contributed 10, which is a third of all deaths in the province. Six maternal death notification forms from Hwange district that were sent to the provincial office were not accompanied by maternal death audit reports as per requirement. Timeliness of the maternal death notification forms reaching the provincial office was also a challenge. For two maternal deaths that occurred in Hwange in February and May 2016, the forms only reached the provincial office in September 2016 meaning that the forms were 7and 4 months late respectively. Deaths that are notified very late are often missed by the surveillance system and missing maternal deaths creates a potential for seriously underestimating the magnitude of maternal mortality within facilities. Information from the MDSR system is important in refining, targeting and ensuring efficient allocation of resources in the fight against maternal mortality. The study was conducted to evaluate the MDSRin Hwange district in 2017.Specifically, we assessed health worker knowledge on MDSR, assessed the systems’ usefulness and its attributes. We also determined reasons for late notification of maternal deaths in Hwange District, 2017.

## Methods

### Study design

We conducted a descriptive cross-sectional study using the updated US Centers for Disease Control and Prevention guidelines for surveillance system evaluation in 2017 [14].

### Study setting

Hwange is one of the seven districts in Matabeleland North province serviced by 22 health facilities, which include four hospitals, two urban clinics, 13 rural health facilities and three private surgeries. The district is situated in the south-western part of Zimbabwe, and mining and tourism activities dominate it. According to the Zimbabwe population census of 2012, the population of Hwange district is 144,745.

### Study population

The Provincial Maternal and Child Health Officer, Medical Superintendent, District Medical Officer, Community Sister/District Nursing Officer were the key informants in the study because they have in depth knowledge of what is going on in the community with regards to maternal health. The PMCHO is the overall accounting officer in the province as far as maternal health is concerned. The Medical Superintendent is the overseer at Lukosi Rural hospital and communicates directly with the PMCHO. At district level the DMO is in charge of the district. The Community sister/ District Nursing Officer provides an entry point in terms of communication regarding maternal deaths from the Rural Health Centres. At facility level it is the responsibility of the attending nurse to fill the Maternal Death Notification Form (MDNF) and reporting to the next level. Doctors, Nurses/Midwives and Primary Care Nurses were the primary respondents. All (*n* = 10) maternal death notification forms for 2016 were also reviewed. Maternal death audit reports were also reviewed.

### Sample size calculation

We used the Dobson’s formula for calculating sample size for single proportions to calculate sample size.:

$$ \mathrm{n}=\frac{{\mathrm{z}}^2\times \mathrm{p}\left(1\hbox{-} \mathrm{p}\right)}{{\mathrm{d}}^2}. $$

where n = required sample size.

p = proportion of health workers with knowledge on MDSR.

d = margin for error i.e. 10%.

Assuming that the timeliness of maternal deaths notified in less than 24 h from the occurrence of the death is 90% and a 10% attrition rate, a minimum sample size of 39 health care workers were interviewed. Due to the paucity of information on MDSR locally the sample size was calculated using a study by Urquiza et al. on MDSR in Mexico [15].

### Sampling procedure

Hwange district has a total 22 health facilities that offer maternal services. Four of these facilities are hospitals which were purposively selected for the study because they provide different hospital settings (urban government hospital, rural government, mission hospital and a private hospital). Two facilities were urban clinics, of which only one was randomly selected. Four facilities were randomly selected from the rural district council clinics. The same method was used to select other health facilities. A total of 11 facilities participated in the study; at least two health workers from these facilities who were found on duty on the day of the interview were interviewed. A minimum of eight health workers in the maternity department on duty on the day of the interview were recruited into the study from the district hospital. From the mission hospital, seven health workers on duty at the maternity department were also interviewed. From the remaining two private hospitals five health workers on duty were interviewed in each hospital. Some of these facilities experienced maternal deaths during the period under review, these included Victoria Falls hospital, Lukosi Rural Hospital and Hwange Colliery hospital.

### Data collection techniques

We collected information on knowledge levels of health care workers on MDSR, assessed usefulness and attributes of the MDSR. Data for the study was collected by the researcher using paper based interviewer administered questionnaires. It took on average about 15 to 20 min to administer the questionnaire. Data was entered manually. 112A checklist for resource availability was used to assess the stability of the system. Maternal death notification forms of all the 10 deaths that occurred in Hwange District in 2016 were reviewed.

### Pretesting of data collection tools

We pretested our data collection tools at Tsholotsho Hospital, because the hospital provided a similar setting with the area under study. The sequence of the questions in the questionnaire was altered so that the questions followed a logical sequence that made sense to respondents.

### Data analysis

We used Epi Info version 7 to compute frequencies, means and proportions.

### Usefulness of the surveillance system

According to the updated CDC guidelines for evaluation of surveillance systems, a public health surveillance system is useful if it contributes to the prevention and control of adverse health-related events, including an improved understanding of the implication of such events (15). Respondents were asked what the data collected on maternal deaths was used for, and also what public health actions were taken based on the surveillance data. Minutes and reports of meetings held on MDSR were produced as evidence.

### System attributes

The definitions used for all the attributes assessed are from the CDC.

### Simplicity

CDC defines simplicity of a public health surveillance system as both its structure and ease of operation while still meeting their objectives. This attribute was assessed by determining whether the implementers of the system have ever filled any MDSR forms and also if they felt there is a need for them to be further trained in the exercise.

### Acceptability

Acceptability is the willingness of persons and organisations to participate in a surveillance system. Health care workers were asked whether they were willing to continue participating in the MDSR. In addition, completeness and timeliness will also be assessed as a proxy of acceptability.

### Stability

Stability is the reliability and availability of the surveillance system. Reliability is the ability to collect, manage and provide data properly without failure. Availability is the ability of a surveillance system to be operational when it is needed. Stability of the MDSR was assessed by checking for consistency in reporting, availability of communication equipment and other material resources needed for the surveillance system.

### Timeliness

Timeliness refers to the speed at which data is transmitted between different levels in the surveillance system. It was assessed by checking whether MDNF are completed within seven days of a maternal death and are then sent to the Provincial Maternal and Child Health Officer within 14 days of the maternal days as stipulated.

### Data quality

The quality of data is influenced by the clarity of surveillance forms, the quality of training and the supervision of persons who complete the maternal death notification forms and the amount of care that is exercised in managing the surveillance data. A review of these features of a surveillance system provided an indirect measure of the quality of data. MDNF were reviewed to check for completeness of the notification forms. Also, the qualifications of the people who conduct maternal death audits were checked.

### Sensitivity

The sensitivity of a surveillance system can be assessed by the proportion of cases detected by the surveillance system. This attribute was assessed by asking key respondents the number of maternal deaths that were picked by the MDSR system, through verifying whether maternal deaths were correctly classified.

A 1–5 Likert scale was used to measure the level of knowledge among healthcare workers. Good was a score of 4–5, fair being a score of 3 and poor for a 0–2 score. Respondents’ knowledge was assessed through being able to correctly define the acronym MDSR, knowledge of the correct reporting timeliness for a maternal death, knowledge of the flow of information in the MDSR system among others. A likert scale was used because it is very quick, easy to run, analyse and draw conclusions based on the responses from respondents.

### Ethical considerations

Permission to carry out the study was sought from the Institutional Review Board of Matabeleland North Province and Health Studies Office. The purpose of the study was explained, and confidentiality assured. Informed written consent with a portion to sign on the questionnaire was sought from study participants and assurance of anonymity and confidentiality was given.

## Results

From the calculated sample size of 39 respondents, we were able to recruit 36 participants for our study yielding a 92.3% response rate. Of the 36 health workers interviewed 26 (72.2%) were female. More than half of the respondents were midwives 20 (55.4%), and the other health workers contributed 16 (44.6%). The majority of the respondents 20 (55.6%) were working in government health facilities while rural district council facilities provided 9 (25.0%) and the rest 7 (19.4%) were from the mission, private and urban local authorities. The median years of service for respondents were 10 years (Q1 = 8 years, Q_3_ = 11.5 years) while the median age of respondents was 36 years (Q1 = 34 years, Q_3_ = 40 years) [Table 1].

**Table 1:** Demographic characteristics of Health Workers in Hwange District, 2017

Variable	Category	Frequency *n* = 36 (%)
Sex	Females	26 (72.2)
Designation	Doctor	1 (2.8)
	Midwife	20 (55.6)
	Registered General Nurse	2 (5.6)
	Primary Care Nurse	12 (33.3)
	Other	1 (2.8)
Type of facility	Government	20 (55.6)
	Mission	3 (8.3)
	Rural District Council	9 (25.0)
	Urban Local Authority	1 (2.8)
	Private	3 (8.3)

Median age of respondents36 years (Q_1_ = 34 years: Q_3_ = 40 years)

Median years in service10 years (Q_1_ = 8 years: Q_3_ = 11.5 years)

### Reasons for late notification of maternal deaths

Poor health worker knowledge on the maternal death surveillance and response system was cited by 24 (66.7%) of the respondents. Half of the respondents 18 (50%) also highlighted that the lack of training on the MDSR system was another reason for late notification of maternal deaths in the district. Unavailability of guidelines and maternal death notification forms as well as lack of knowledge on the flow of information in the surveillance system were reported as other reasons by 10 (27.8%) and 12 (33.3%) respectively [Table 2]. Information on the lack of guidelines at the facilities was collaborated by the PMCHO. Triangulation of information regarding the availability of guidelines was done through key informants at district level.

**Table 2:** Reasons for late notification of maternal deaths, Hwange District, 2017

Reasons	Frequency *n* = 36 (%)
Inadequate health worker knowledge of the MDSR system	24 (66.7)
Lack of training on the surveillance system	18 (50)
Lack of knowledge on the flow of information in the surveillance system	12 (33.3)
Unavailability of guidelines	10 (27.8)
Unavailability of investigation/notification forms	10 (27.8)
Inadequate human resources	9 (25.0)
Misclassification on the cause of death	7 (19.4)

### Knowledge of the surveillance system

The majority of respondents 23 (63.9%) reported that they have never notified a maternal death before. The notification of a maternal death is the responsibility of the attending nurse. Thirty–three (94.3%) of respondents were able to state that a maternal death should be reported within 24 h to the next level. Half of the respondents 17 (47.2%) correctly reported that three maternal death notification forms (MDNF) are completed when notifying a maternal death. The majority of respondents 34 (94.4%) reported that an MDNF should be completed within 7 days of the occurrence of a maternal death. The overall knowledge of health workers was fair.

### Usefulness of the MDSR system

Almost all the respondents 33 (91.7%) confirmed that MDSR data was being used at their level. The majority of the respondents, 22 (61.1%) reported that they use MDSR data to provide better information for action and monitoring improvements in maternal health while 20 (55.6%) used the MDSR data to quantify and determine the causes and avoidance of maternal deaths in the district. A majority of respondents 31 (86.1%) reported that they had taken decisions based on MDSR data (Table 3). There was evidence of utilisation of MDSR data in the district; facilities were able to produce minutes and maternal death audit reports.

**Table 3:** Usefulness of the Maternal Death Surveillance and Response system, Hwange District, 2017

Variable	Category	Frequency
Use of MDSR data	Promoting routine identification of maternal deaths	5 (13.9%)
	Promoting timely notification of maternal deaths	13 (36.1%)
	Linking health information systems and quality improvement processes from local to national level	11 (30.6%)
	Quantify and determine causes of maternal deaths	20 (55.6%)
	Strengthening vital registration	3 (8.3%)
	Providing better information for action and monitoring improvements in maternal health	22 (61.1%)

On action taken based on MDSR data 26 (72.2%) of the respondents mentioned that they used MDSR data for strengthening health care system in early identification of at risk and refer them urgently. Also, 21 (58.8%) stated that they use data for increasing community and institutional awareness of maternal mortality. Respondents 33 (91.7%) were of the opinion that the MDSR system is useful in Hwange District [Table 3]. The usefulness of the MDSR system was evident as the facilities had charts and graphs stuck on the walls for all to see, the charts and graphs were for all facilities seen, they were displaying data on maternal health e.g. number of live births recorded at the facility, maternal deaths recorded at the same facility in a given time frame. These charts were targeting health care workers, the local community as well as anyone interested in maternal health.

### Simplicity of the MDSR system

Only 13 (36.1%) respondents had previously completed an MDNF. Out of these nine had never faced challenges in completing the MDNF. The majority of the respondents stated that they usually took more than 15 min to complete the MDNF. Almost all the respondents 33 (91.7%) felt that there was a need for training in the completion of the MDNF.

### Acceptability of the MDSR system

All participants felt that it was their responsibility to complete the MDNF and they were willing to continue participating in the MDSR system. Almost 78% (*n* = 28) reported that MDSR data was analysed and 15 (55.6%) were able to produce reports as evidence of data analysis. Twenty-one (60.0%) reported having held maternal death audit meetings at their facilities. Reports on the audit meetings held at facilities were availed.

### Stability of the MDSR system

The majority of respondents 30 (83.3%) interviewed were not trained in MDSR, only six reported having received training. Those that were trained received training in the form of on the job training and workshop training. Five out of eleven health facilities had maternal death notification forms, and they were adequate. Only two facilities out of 11 (18.2%) had maternal death case definitions, and the definitions were kept on a shelf and not displayed. All 11/11 (100%) facilities had a phone for communication with next level of care. Four out of 11(36.4%) health facilities were found with maternal death audit guidelines [Table 4].

**Table 4:** Resources needed to run the MDSR system**,** Hwange District, 2017

Resources	Frequency *n* = 11 (%)
Facility mobile phone	8 (72.7)
Maternal death notification forms	5 (45.5)
Maternal death case definitions	2 (18.2)
Guidelines for maternal death audits	4 (36.4)
Computer and printer	10 (90.9)
Internet connectivity	4 (36.4)

The cost of completing three MDNF and submitting them to the district office was calculated. Assuming that the health worker completing the form earned a monthly salary of $500, twenty-two working days per month and an eight-hour working day. The cost of transporting an MDNF @ $0.31/km was $57.04. We used the furthest health facility from the district office (Kamativi clinic). The total cost of running the MDSR system in Hwange district was $60.78 [Table 5].

**Table 5:** Cost of running an MDSR system in Hwange District, 2017

Item	Unit cost	Multiplying factor	Total
Completing the Maternal Death Notification Form	$2.84	1 h	$2.84
Stationery	$0.30	3 copies per MDNF	$0.90
Transport	$0.31/km	184 km	$57.04
Total			**$60.78**

## Discussion

All the deaths recorded in the district were institutional deaths, no community deaths were recorded. This could be as a result of lack of a functional system for routinely identifying maternal deaths in the community. There is a likelihood of deaths occurring in the community but because there is no way of capturing and recording these deaths, they may be going unnoticed. This important finding concurs with findings made by Moodley et.al in South Africa where they emphasised that maternal death enquiries are facility based with no system currently for routinely identifying deaths in the community [16]. Our findings are in contradiction with the WHO’s recommendations on community participation in MDSR which emphasises the importance of sharing information on pregnancy related deaths with communities including discussion of different factors causing these deaths and affecting access to skilled care [17].

Lack of information sharing between health care workers and communities creates a huge gap and subsequently pregnancy related issues affecting the same communities are not given the attention they deserve. Another study done in Sierra Leone revealed that at community level the notification and reporting of maternal deaths was one of the biggest challenges identified. The community failed to report maternal deaths because of numerous socio cultural reasons. The study also revealed that maternal deaths did occur but were not reported because of fear of repercussions by community members. This might also be one of the contributing factors for not recording community deaths in Hwange district.

Our study results revealed that health workers in Hwange district had fair knowledge on the MDSR. Lack of adequate knowledge on the MDSR system meant that health workers had little idea of what was expected from them. This gap in knowledge fuels further delays in the maternal death notification process. Furthermore, lack of adequate knowledge on the MDSR allows for many deaths, maternal or otherwise to go unrecorded or are incorrectly classified which results in underreporting of maternal mortality. Every misclassified or unrecorded maternal death is a lost opportunity to take corrective action to ensure that other women do not die in the same way [18].

Our study also found some inconsistencies from health workers with regards to the number of maternal death notification forms that are completed when notifying a maternal death, half of the health workers correctly mentioned three forms, and the other reported that four maternal death notification forms are completed. Such inconsistencies are a clear indication that health workers in the district lack adequate knowledge of the surveillance system. The inconsistencies can be attributed to the fact that most of the maternal deaths recorded occurred in the hospital setting and the notification process was only confined to those health workers in the district hospital thereby excluding those health workers in the rural health facilities. These findings are consistent with findings made by Mutsigiri at.al in Mutare district where they reported that most maternal deaths occurred at the district or provincial hospitals hence only health workers at these referral facilities were exposed to the surveillance system and thereby excluding those at the rural health facilities [19].

We also found that health workers were able to correctly state the correct timelines for the notification of a maternal death to the next level. However, even with this knowledge of timelines, Hwange district was notifying maternal deaths late. Lack of training on the surveillance system coupled with the unavailability of notification forms at the health facilities were reasons cited by most health workers for notifying maternal deaths late. Inadequate human resources were also cited as another reason for late notification of maternal deaths in Hwange district. The district had three doctors practicing however we managed to recruit only one medical doctor, this is because the district is seriously short staffed and the doctor was the only one available at the time of data collection. Similar findings were also made by Mutsigiri et al., where they highlighted that maternal deaths were not reported on time because health workers encountered too much workload and a shortage of staff. Timely reporting of a maternal death improves the accuracy of information obtained and underscores the benefits of making a maternal death a notifiable event [18]. Timely notification of maternal deaths, assessment and confirmation of cases, increased awareness and advocacy adds value to active surveillance of maternal deaths [18].

Our study revealed that there was a need for training of healthcare workers in the completion of the MDNF, the majority of the health workers had never completed an MDNF before therefore respondents felt that they were inadequately prepared in the event a maternal death occurs at their facility. This training gap is because in rural health facilities maternal deaths are rare events so this explains why most health workers have never completed an MDNF before. These findings are consistent with findings made by Chirundu et al. (2017) in Sanyati district where they reported that only 24% of respondents reported having completed an MDNF before. Training of health workers in MDSR give them a practical approach to maternal health and also boost their knowledge of the MDSR system. This training of health care workers on MDSR will, therefore, equip them to have a better reporting system which will capture critical information that will inform targeted approaches to improve maternal health care [20]. The knowledge gap fuels underreporting and poor use of MDSR data, this, in turn, makes data inappropriate for proactive response, planning and resource allocation [21].

We also found that facilities in Hwange had no maternal death case definitions and the few that had the case definitions kept them on the shelves, and they were not displayed for all to see and use. Keeping maternal case definitions on the shelves may be a sign that these definitions are not being used at all in these facilities. Case definitions act as refresher material for health workers, and they aid in the proper classification of a maternal death when it happens. These definitions are part of a strategy for the improvement of the quality of maternal health care, which will ideally, lead to a reduction of maternal morbidity and mortality [20].

The usefulness of the MDSR system was evident in Hwange district; there was evidence of the use of data on the surveillance system through the production of facility reports as well as wall charts with information such as graphs and statistics. The acceptability of the MDSR system among health workers was reportedly high; this is however not a true reflection of the actual performance of the MDSR system in Hwange. Health workers are aware of their daily responsibilities and their mandate to report maternal deaths on time, so many of them are likely to report a willingness to continue participating in the MDSR system.

The stability of the MDSR system was affected by lack of training and or induction of health workers on the surveillance system, unavailability of case definitions, unavailability of the notification forms as well as unavailability of maternal death audit guidelines at facilities. These resources when in short supply trigger the inability of health systems to measure levels and trends in maternal mortality reliably, this contributes to lack of accountability and in turn lack of progress in achieving the goal of reducing maternal mortality through the implementation of the MDSR system [22]. Almost all facilities had a functional computer and a printer for producing material that is needed in the surveillance system. However very few of the institutions had an internet connection. Facilities ought to have access to technology so that health workers can access information regarding maternal health whenever they need it.

Our study had some limitations. Most of the information obtained was subjectively assessed; however, some evidence to support the findings was obtained from the facility records (reports and minutes). We were also unable to examine all the system attributes due to non availability of data on the attributes. However we objectively assessed some of the attributes hence this study provides useful information on the performance of the maternal death surveillance and response system in Hwange district.

## Conclusions

We concluded that in Hwange district, the MDSR system is useful, acceptable, flexible and somewhat stable. The system is however costly and not simple to use by health workers. Reasons for late notification of maternal deaths were lack of adequate knowledge of health care workers on the MDSR system and lack of proper induction on the MDSR in the district. We recommend induction or orientation of all health workers involved in MDSR, Standard Operating Procedures on the completion of maternal death notification forms to be availed to health workers in health facilities. As a result of this study, a soft copy of the latest maternal death notification form was distributed to those health facilities that did not have an MDNF. Also, copies of the maternal death audit guidelines were sourced and distributed to all health facilities.

## Abbreviations

CDC: Centers for Disease Control and Prevention
DHIS 2: District Health Information System 2
MDG: Millennium Development Goals
MDNF: Maternal Death Notification Form
MDSR: Maternal Death Surveillance and Response
MMSR: Maternal Mortality Surveillance and Response
WHO: World Health Organisation
